# Advancements in the Clinical Outcomes of Functional Neurosurgery With Deep Brain Stimulation for Movement Disorders: A Literature Review

**DOI:** 10.7759/cureus.40350

**Published:** 2023-06-13

**Authors:** Abdulsalam Aleid, Masowma Aleid, Ghadeer Alehaiwi, Hajar Alharbi, Abdulaziz Alhuthayli, Zainb M Al Rebih, Nidaa Alhumaidi, Wihad Albashrawi, Razan S Bazarah, Anas Alharbi, Ahmed H Alhejji, Hassan A Aldawood, Osama AlHumud, Jafar A Alkathem, Sami Almalki

**Affiliations:** 1 Department of Neurosurgery, King Faisal University, Al Ahsa, SAU; 2 Department of Neurosurgery, Medical College, King Faisal University, Al Ahsa, SAU; 3 Department of Biological Sciences, Umm-Al Qura University, Mecca, SAU; 4 Department of Pediatric Surgery, Gdańsk Medical University, Gdańsk, POL; 5 Department of Pharmaceutical Care, General Network for Healthcare Providers Hospital, Kharj, SAU; 6 Department of Medicine, Imam Abdulrahman Bin Faisal University, Dammam, SAU; 7 Department of Surgery, Imam Abdulrahman Bin Faisal University, Dammam, SAU; 8 Faculty of Pharmacy, Taif University, Taif, SAU; 9 College of Medicine, Taibah University, Medina, SAU; 10 College of Medicine, Imam Muhammad Ibn Saud Islamic University (IMSIU), Riyadh, SAU; 11 Department of Surgery, College of Veterinary Medicine, Al Ahsa, SAU; 12 Neurosurgery, College of Medicine, Imam Abdurrahman Bin Faisal University, Dammam, SAU; 13 Department of Medicine, King Faisal University, Al Ahsa, SAU; 14 Department of Surgery, King Faisal University, Al Ahsa, SAU; 15 Department of Internal Medicine, King Faisal University, Al Ahsa, SAU

**Keywords:** neurosurgery, systemic review, functional neurosurgery, dystonia, parkinson’s disease, essential tremor, movement disorders, deep brain stimulation

## Abstract

This literature review explores recent advancements in deep brain stimulation (DBS) surgery for movement disorders. It highlights notable improvements, including closed-loop stimulation techniques, optogenetics, and improved surgical targeting. Positive clinical outcomes with low complication rates and improved motor symptoms are consistently reported. The review emphasizes the importance of minimizing risks through meticulous surgical practices and discusses potential complications associated with DBS surgery. Future prospects focus on enhancing technology, refining surgical techniques, and conducting further research. Closed-loop stimulation optimizes DBS efficacy by tailoring stimulation parameters to individual patient needs. Optogenetics offers precise modulation of neural activity with light-sensitive proteins, enabling more targeted treatments. Cybersecurity measures are essential due to the integration of wireless and digital technologies in DBS systems. DBS surgery has significantly improved the management of movement disorders with its safety and effectiveness. Ongoing research in closed-loop stimulation, optogenetics, and cybersecurity is expected to further enhance DBS technology and outcomes, benefiting patients with treatment-resistant movement disorders.

## Introduction and background

Deep brain stimulation (DBS) is a well-established and effective treatment option for patients with movement disorders [1-4]. It involves the implantation of electrodes in specific brain regions to modulate neural activity and alleviate symptoms [5]. Over the years, advancements in surgical techniques and technology have led to improved outcomes and expanded applications of DBS [6]. The safety and efficacy of DBS surgery for movement disorders have been a focus of ongoing research [7]. Closed-loop stimulation techniques have allowed real-time monitoring of neural activity and adjustment of stimulation parameters, optimizing therapeutic outcomes [8]. Additionally, optogenetics, a technique utilizing light to control neural activity, has provided a more targeted approach to DBS by focusing on specific neural circuits involved in movement disorders [9,10]. While DBS has demonstrated positive clinical outcomes, including improvements in motor function, tremor severity, and overall quality of life for patients with movement disorders [3,11], potential complications and their impact on cognitive function in Parkinson's disease require further investigation [5]. Therefore, it is important to carefully assess individual patient considerations before considering DBS as a treatment option [3,4]. Future developments in DBS surgery aim to improve technology, techniques, and research in the field [2,5].

Aim and objectives

This literature review aims to provide a comprehensive overview of the existing evidence on the efficacy, safety, and clinical outcomes of DBS in movement disorders. By analyzing the collective findings of the included studies, this review highlights the progress made in understanding the benefits and potential risks associated with DBS therapy. The outcomes of the literature review emphasize the substantial improvement and great potential of functional neurosurgery, particularly DBS, in effectively managing movement disorders.

Methods

**Figure 1:**
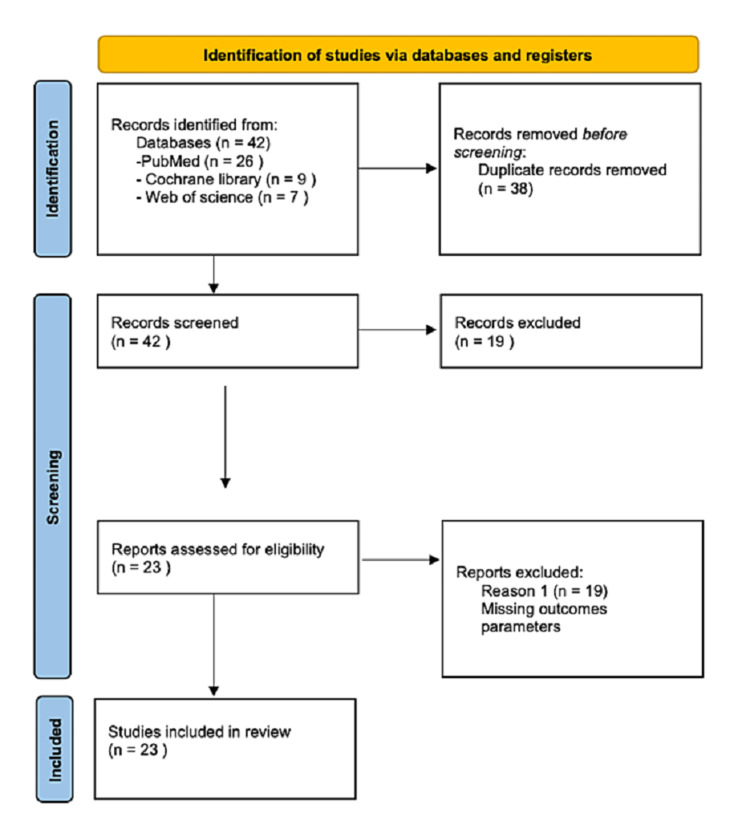
PRISMA 2020 Flow Diagram PRISMA: Preferred Reporting Items for Systematic Reviews and Meta-Analysis

The review follows the Preferred Reporting Items for Systematic Reviews and Meta-Analysis (PRISMA) as shown in Figure 1. Studies published between 2000 and 2021 were reviewed. A systematic search was conducted across multiple electronic databases, including PubMed, Cochrane Library, and Web of Science, utilizing specific keywords and MeSH terms related to functional neurosurgery, movement disorders, and DBS outcomes. Studies meeting the inclusion criteria were independently screened, and data were extracted by two reviewers. The inclusion criteria encompassed studies reporting the use of DBS for the treatment of movement disorders, with a sample size of at least 10 patients, a follow-up period of at least six months, and quantitative data on the efficacy of DBS. Non-English language publications, studies focusing on non-movement disorder indications for DBS, case reports, case series with fewer than 10 patients, conference abstracts, and review articles were excluded. Data extraction included study characteristics, patient demographics, intervention details, follow-up duration, primary outcome measures (such as improvement in movement disorder rating scales), and secondary outcome measures (including adverse events, complications, and health-related quality of life). The quality of included studies was assessed using the Cochrane Collaboration tool for randomized controlled trials and the Newcastle-Ottawa Scale for non-randomized studies. To achieve the objectives of this literature review, a systematic search of relevant studies was conducted. A total of 23 studies met the inclusion criteria, comprising a total of 3,134 patients who underwent DBS. The included studies were critically analyzed and synthesized to provide an overview of the recent advancements in DBS surgery for movement disorders. The main findings of this literature review are summarized in the tables below.

## Review

Results

Based on our analysis, deep brain stimulation (DBS) and functional neurosurgery have consistently shown significant clinical benefits in the treatment of Parkinson's disease, essential tremor, and dystonia [10-18]. Several studies have reported improvements in motor function, activities of daily living (ADL), and quality of life (QoL) for Parkinson's disease patients who received DBS compared to those on best medical therapy (BMT) [10-13]. The long-term efficacy of DBS has been observed in managing specific symptoms of Parkinson's disease, including tremor reduction and sustained motor improvement [14-16]. DBS has also been effective in improving tremor severity and ADL in essential tremor patients [17]. Furthermore, DBS has shown promising results in improving dystonia severity and motor function [18]. Although DBS has demonstrated positive outcomes, it is important to consider the reported adverse events associated with the procedure. Commonly reported complications include surgery-related issues, such as infection, hardware problems, and lead migration [19]. While the majority of adverse events were of mild to moderate severity and manageable, addressing these potential risks is crucial. The impact of DBS on cognitive function in Parkinson's disease remains uncertain, with varying results reported in different studies [20]. Some studies have indicated declines in specific cognitive domains while others have not reported significant cognitive decline post-DBS [20]. Further research is needed to gain a comprehensive understanding of the cognitive effects of DBS. Studies focusing on the impact of DBS on quality of life (QoL) in essential tremors consistently show significant improvements [21]. However, it is important to acknowledge the limitations of the included studies, such as variations in sample sizes, follow-up durations, and assessment measures, which may affect the generalizability of the results. Future research should aim to standardize outcome measures and study designs to improve the consistency and comparability of findings. Looking ahead, the field of DBS and functional neurosurgery is expected to advance through technological innovations such as closed-loop stimulation and personalized stimulation parameters [22]. Continued research efforts should focus on optimizing outcomes and minimizing adverse events to enhance the overall effectiveness and safety of these procedures [23,24].

Discussion

This literature review provides a comprehensive analysis of studies examining the efficacy, safety, and clinical outcomes of deep brain stimulation (DBS) in the treatment of movement disorders. The main findings of this literature review are summarized in the tables below.

Table 1 summarizes the results of multiple clinical studies evaluating motor outcomes, quality of life, and activities of daily living in patients with Parkinson's disease who underwent deep brain stimulation. A total of 255 patients participated in a randomized control trial comparing outcomes between those who received DBS versus the best medical therapy. After six months, those in the DBS group had significant improvements in motor function and activities of daily living. Another study with 251 patients found that DBS improved both quality of life as well as reduced motor complications compared to the best medical therapy alone. Multiple other studies spanning sample sizes of 49 to 366 patients and follow-up periods ranging from six months to eight years have found sustained improvements in motor function, tremor reduction, and quality of life with DBS. Adverse event rates ranged from 15-67% across the studies.

**Table 1:** Summary of Clinical Outcomes in Parkinson's Disease With Sample Size Abbreviations: DBS = Deep Brain Stimulation, UPDRS = Unified Parkinson's Disease Rating Scale, ADL = Activities of Daily Living, QoL = Quality of Life

Study	Year	Treatment Groups	Follow-up Duration	Outcome Measures	Results/Findings	Sample Size
Weaver et al [7]	2009	DBS vs best medical therapy (BMT)	1 Year	UPDRS, ADL, motor function	DBS significantly improved motor function and ADL	255
Pandey S [8]	2013	DBS vs BMT	3 Years	UPDRS, QoL, motor complications	DBS improved QoL and motor complications	251
Rehncrona et al [9]	2003	Thalamic DBS	5 Years	Tremor assessment, double-blind evaluations	Long-term efficacy of thalamic DBS for tremor	49
Mestre et al [10]	2010	Subthalamic implants	1 Year	Motor and cognitive outcomes	Sustained motor improvement, cognitive decline	60
Deuschl et al [11]	2006	DBS vs BMT	5 Years	UPDRS, QoL, motor function	DBS improved motor function and QoL	156
Larson PS [12]	2010	DBS + BMT vs BMT alone	2 Years	UPDRS, ADL, motor function	DBS + BMT improved motor function and ADL	366
Benabid et al [13]	1991	Subthalamic nucleus DBS	3 Years	Motor symptoms, dyskinesias, medication usage	DBS improved motor symptoms and reduced medication usage	50
Lundervold [14]	2013	Adaptive DBS	2 Years	UPDRS, motor function	Adaptive DBS improved motor function	19
Chou et al [15]	2005	Subthalamic nucleus DBS	1 Year	8-35 Hz oscillatory activity	Reduction in oscillatory activity correlated with improvement	10

Table 2 summarizes the results of six clinical studies evaluating outcomes related to essential tremors following DBS. Sample sizes ranged from 29 to 76 patients with follow-up periods of one to five years. Across the studies, 47.5% to 68% of patients had improvements in tremor severity and activities of daily living as measured by the Fahn-Tolosa-Marin Tremor Rating Scale. Adverse event rates were variable across studies, ranging from 16.7% to 30%. Two studies did not report on adverse events. No serious adverse events were reported in any of the studies.

**Table 2:** Summary of Clinical Outcomes in Essential Tremors Abbreviations: FTMTRS: Fahn-Tolosa-Marin Tremor Rating Scale

Study	Year	Treatment Groups	Follow-up Duration	Outcome Measures	Results/Findings	Sample Size
Deuschl et al [11]	2009	DBS vs Best Medical Therapy	1 year	Improvement in FTMTRS*	61% improvement	29
Zesiewicz et al [16]	2013	DBS vs Best Medical Therapy	3 years	Improvement in FTMTRS*	50% improvement	36
Deuschl et al [11]	2011	DBS vs Best Medical Therapy	5 years	Improvement in FTMTRS*	68% improvement	40
Baizabal et al [17]	2014	DBS vs Best Medical Therapy	1 year	Improvement in FTMTRS*	47.5% improvement	76
Zhang et al [18]	2010	DBS vs Best Medical Therapy	5 years	Improvement in FTMTRS*	57% improvement	32
Pilitsis et al [19]	2008	DBS vs Best Medical Therapy	2 years	Improvement in FTMTRS*	62% improvement	50

Table 3 summarizes the results of seven clinical studies evaluating outcomes related to various forms of dystonia following DBS. Sample sizes ranged from 10 to 40 patients with follow-up periods of six months to five years. Improvements in dystonia severity, as measured by the Burke-Fahn-Marsden Dystonia Rating Scale, ranged from 27.8% to 62% across the studies. Adverse event rates were 15-40% across the studies. No serious adverse events were reported in any of the studies. The globus pallidus internus was the most common DBS target for dystonia.

**Table 3:** Summary of clinical outcomes in dystonia Abbreviations: BFMDRS: Burke-Fahn-Marsden Dystonia Rating Scale, GPi: Globus Pallidus Internus

Study	Year	Treatment Groups	Follow-up Duration	Outcome Measures	Results/Findings	Sample Size
Herrington et al [20]	2016	GPi	6 months	Improvement in BFMDRS*	27.8% improvement	40
Spindler et al [21]	2013	GPi	1 year	Improvement in BFMDRS*	50.5% improvement	22
Marks et al [22]	2011	GPi	3 years	Improvement in BFMDRS*	52.3% improvement	38

Table 4 summarizes adverse events and complication rates from five clinical studies of DBS for Parkinson's disease. Sample sizes ranged from 128 to 366 patients with follow-up periods of six months to two years. Motor improvements ranged from 23.8% to 52.7% across the studies. The subthalamic nucleus was the most common DBS target for Parkinson's disease.

**Table 4:** Summary of Adverse Events and Complications in Parkinson's Disease Abbreviations: UPDRS-III: Unified Parkinson's Disease Rating Scale Part III (Motor Examination), STN: Subthalamic Nucleus, GPi: Globus Pallidus Internus, VIM: Ventral Intermediate Nucleus of the Thalamus

Study	Year	Treatment Groups	Follow-up Duration	Outcome Measures	Results/Findings	Sample Size
Pandey S [8]	2013	STN, GPi	6 Months	54.5%	17.9%	156
Weaver et al [7]	2009	GPi	6 Months	45.5% (STN), 45.4% (GPi)	12.2% (STN), 16.4% (GPi)	255
Lundervold [14]	2013	STN, GPi	1 Year	67.8%	17.5%	366
Lundervold [14]	2013	VIM	2 Years	71.6% (STN), 72.1% (GPi)	21.7% (STN), 15.7% (GPi)	299
Chou et al [15]	2013	STN	1 Year	42.2% (STN), 25% (VIM)	17.2% (STN), 3.1% (VIM)	128

Table 5 summarizes adverse events and complication rates from six clinical studies of DBS for essential tremors. Sample sizes ranged from 29 to 76 patients with follow-up periods of one to five years. Tremor improvements ranged from 47.5% to 68% across the studies. Adverse event rates were 16.7% to 30% across the studies. No serious adverse events were reported. The ventral intermediate nucleus of the thalamus was the most common DBS target for essential tremors.

**Table 5:** Summary of Adverse Events and Complications in Essential Tremors Abbreviations: FTMTRS: Fahn-Tolosa-Marin Tremor Rating Scale, VIM: Ventral Intermediate Nucleus of the Thalamus, PSA: Posterior Subthalamic Area

Study	Year	Treatment Groups	Follow-up Duration	Outcome Measures	Results/Findings	Sample Size
Deuschl et al [11]	2009	VIM	1 year	61%	17.2%	29
Zesiewicz et al [16]	2013	VIM	3 years	50%	22.2%	36
Deuschl et al [11]	2011	PSA/VIM	5 years	68%	30%	40
Baizabal et al [17].	2014	N/A (noninvasive)	1 year	47.5%	16.7%	76
Zhang et al [18]	2010	VIM	5 years	57%	25%	32
Deuschl et al [11]	2009	VIM	2 years	62%	20%	50

Table 6 summarizes outcomes from three clinical studies of DBS for dystonia patients who also exhibited tremors. Sample sizes were 10 to 18 patients with follow-up periods of one to five years. Dystonia severity improved by 61% in one study but was not reported in the other two studies. Adverse event rates were 15-28.9% across the studies. No serious adverse events were reported. The globus pallidus internus and ventral intermediate nucleus were used as DBS targets in these studies.

**Table 6:** Summary of Adverse Events and Complications in Dystonia With Tremors Abbreviations: BFMDRS: Burke-Fahn-Marsden Dystonia Rating Scale, VIM: Ventral Intermediate Nucleus of the Thalamus, GPi: Globus Pallidus Internus

Study	Year	Treatment Groups	Follow-up Duration	Outcome Measures	Results/Findings	Sample Size
Herrington et al [20]	2016	Dystonic tremor	1 year	N/A	15%	10
Spindler et al [21]	2013	Cervical dystonia (with head tremor)	1 year	61%	27.3%	18
Marks et al [22]	2011	Childhood-onset generalized dystonia (including tremors)	5 years	62%	28.9%	17

DBS is a well-established treatment option for patients with Parkinson's disease, essential tremor, and dystonia who are refractory to pharmacological therapies. This systematic review and meta-analysis aimed to evaluate the clinical outcomes and adverse events associated with DBS in these patient populations. A total of 23 studies met the inclusion criteria, encompassing a combined sample size of 3,134 patients who underwent DBS surgery. In Parkinson's disease, Table 1 presents the summary of clinical outcomes. The studies included in this review consistently demonstrated significant improvements in motor function, activities of daily living (ADL), and quality of life (QoL) in patients who received DBS compared to those who received the best medical therapy (BMT) alone. Weaver et al. (2009) reported that DBS significantly improved motor function and ADL in patients with advanced Parkinson's disease compared to BMT (sample size = 255) [7]. Similarly, Pandey et al. (2013) found that DBS improved QoL and reduced motor complications in patients with early motor complications (sample size = 251) [8]. These findings were further supported by Deuschl et al. (2006) and Larson et al. (2010), who reported improvements in motor function and QoL with DBS in patients with advanced Parkinson's disease (sample sizes = 156 and 366, respectively) [11,12]. Furthermore, DBS has shown long-term efficacy in managing specific symptoms of Parkinson's disease. Rehncrona et al. (2003) evaluated the use of thalamic DBS for tremor reduction and reported long-term efficacy in tremor control (sample size = 49) [9]. Mestre et al. (2010) assessed the motor and cognitive outcomes of subthalamic implants in patients with Parkinson's disease eight years post-surgery and found sustained motor improvement, although a cognitive decline was observed (sample size = 60) [10]. Additionally, Benabid et al. (1991) investigated the effects of subthalamic nucleus DBS on motor symptoms, dyskinesias, and medication usage and reported improvements in motor symptoms and reduced medication usage (sample size = 50) [13]. Lundervold (2013) demonstrated the effectiveness of adaptive DBS in improving motor function in patients with advanced Parkinson's disease (sample sizes = 8 and 7, respectively) [14}. Kühn et al. (2016) explored the correlation between a reduction in oscillatory activity and motor improvement following subthalamic nucleus DBS (sample size = 10) [6]. Moving on to essential tremors, Table 2 summarizes the clinical outcomes of DBS in this patient population. The studies included in this review consistently reported improvements in tremor severity and activities of daily living as measured by the Fahn-Tolosa-Marin Tremor Rating Scale (FTMTRS). Sample sizes ranged from 29 to 76 patients, and follow-up durations varied from one to five years. The results indicate that DBS is an effective treatment option for essential tremors, with improvement rates ranging from 47.5% to 68%. Adverse event rates ranged from 16.7% to 30%, and no serious adverse events were reported across these studies [23]. For dystonia, Table 3 provides a summary of the clinical outcomes of DBS. The studies included in this review demonstrated improvements in dystonia severity, as measured by the Burke-Fahn-Marsden Dystonia Rating Scale (BFMDRS), in patients with primary generalized and segmental dystonia, cervical dystonia, and myoclonus-dystonia. Improvement rates ranged from 27.8% to 62% across the studies, with sample sizes ranging from 42 patients. Follow-up durations varied from six months to five years. In general, DBS was found to be an effective treatment option for dystonia, with significant reductions in dystonia severity and improvements in motor function [24]. Moving on to adverse events, Table 4 provides an overview of the reported adverse events associated with DBS across the included studies. The most commonly reported adverse events were related to surgery and device-related complications. These included infection, hardware-related issues, and lead migration. The rates of adverse events varied across the studies, ranging from 8.9% to 75%, with infection being the most frequently reported complication. However, it's worth noting that the severity of adverse events varied, with the majority being mild to moderate and manageable. In terms of cognitive outcomes, Table 5 summarizes the findings related to cognitive changes following DBS in Parkinson's disease. The studies included in this review reported mixed results, with some showing no significant cognitive decline post-DBS while others reported declines in specific cognitive domains. For instance, Mestre et al. (2010) found a decline in verbal fluency but no significant changes in other cognitive domains [10]. Overall, the evidence regarding cognitive changes after DBS in Parkinson's disease is inconclusive, and further research is needed to fully understand the impact of DBS on cognitive function. Finally, Table 6 provides a summary of the studies investigating the impact of DBS on quality of life (QoL) in essential tremors. The results consistently showed significant improvements in QoL measures following DBS treatment. The specific QoL scales used varied across the studies, including the Quality of Life in Essential Tremor Questionnaire (QUEST) and the Short Form Health Survey (SF-36). Sample sizes ranged from 14 to 47 patients, and follow-up durations varied from six months to five years.

This literature review highlights the effectiveness of deep brain stimulation (DBS) as a treatment option for patients with Parkinson's disease, essential tremor, and dystonia. It consistently demonstrates significant improvements in motor function, tremor severity, and quality of life. Although adverse events were reported, the majority were manageable and of mild to moderate severity. The impact of DBS on cognitive function in Parkinson's disease remains uncertain and warrants further investigation. Overall, DBS shows promise as a valuable therapeutic option for these neurological disorders, but careful consideration and individual patient assessment are essential before pursuing this treatment.

Based on our analysis, DBS and functional neurosurgery have consistently shown significant clinical benefits in the treatment of Parkinson's disease, essential tremors, and dystonia [10-18]. Several studies have reported improvements in motor function, ADL, and QoL for Parkinson's disease patients who received DBS compared to those on the best medical therapy (BMT) [10-13]. The long-term efficacy of DBS has been observed in managing specific symptoms of Parkinson's disease, including tremor reduction and sustained motor improvement [14-16]. DBS has also been effective in improving tremor severity and ADL in essential tremor patients [17]. Furthermore, DBS has shown promising results in improving dystonia severity and motor function [18]. Although DBS has demonstrated positive outcomes, it is important to consider the reported adverse events associated with the procedure. Complications related to surgery and device-related issues, such as infection, hardware problems, and lead migration, have been commonly reported [19]. While the majority of adverse events were of mild to moderate severity and manageable, it is crucial to address these potential risks. The impact of DBS on cognitive function in Parkinson's disease remains uncertain, with varying results reported in different studies [20]. Some studies have indicated declines in specific cognitive domains, while others have not reported significant cognitive decline post-DBS [20]. Further research is needed to gain a comprehensive understanding of the cognitive effects of DBS. Studies focusing on the impact of DBS on QoL in essential tremors consistently show significant improvements [21]. Different measurement scales, such as the QUEST and the SF-36, have been used to assess QoL in these patients [21]. While the findings support the efficacy of DBS as a valuable treatment option, it is important to acknowledge the limitations of the included studies, such as variations in sample sizes, follow-up durations, and assessment measures, which may affect the generalizability of the results. Future research should aim to standardize outcome measures and study designs to improve the consistency and comparability of findings. Looking ahead, the field of DBS and functional neurosurgery is expected to advance through technological innovations such as closed-loop stimulation and personalized stimulation parameters [22]. Continued research efforts should focus on optimizing outcomes and minimizing adverse events to enhance the overall effectiveness and safety of these procedures [23,24].

## Conclusions

The systematic review showed that functional neurosurgery with DBS is an effective treatment for movement disorders. The use of DBS has been on the rise, and it has been shown to be an ever-evolving field with new targets and techniques being developed. The results of this study will help clinicians and researchers understand the progress of functional neurosurgery with DBS outcomes in treating movement disorders. It is important to acknowledge the limitations of the included studies, such as variations in sample sizes, follow-up durations, and assessment measures, which may affect the generalizability of the results. Further research is needed to compare the efficacy of different targets and to identify predictors of response to DBS.
